# Effects of symptom management program on selected health outcomes among older people with chronic obstructive pulmonary disease: a quasi-experimental study

**DOI:** 10.1038/s41598-023-49654-5

**Published:** 2023-12-14

**Authors:** Kannikar Phuthornchai, Samoraphop Banharak, Ladawan Panpanit, Sutin Chanaboon

**Affiliations:** 1 Nong Wua So Hospital, Nong Wua So, Udon Thani Province Thailand; 2 Department of Gerontological Nursing, Faculty of Nursing, Khon Kaen University, Khon Kaen, Thailand; 3 Sirindhorn College of Public Health Khon Kaen, Khon Kaen, Thailand

**Keywords:** Diseases, Health care, Signs and symptoms

## Abstract

Older adults have limitations from their aging process and chronic disease, so developed interventions must pay attention and concern to their aging degeneration and needs. This study aims to study the effects of a symptom management program on selected health outcomes among older people with chronic obstructive pulmonary disease. The quasi-experimental research included the 15 older patients in the control group receiving routine nursing care, while the other 15 in the experimental group received a 4-week symptom management program. First, the general information was analyzed using descriptive statistics. Next, the average health outcomes were analyzed using independent and dependent t-tests, Mann–Whitney U Test, and Wilcoxon Signed Ranks Test. In addition, the readmission rate was compared using Fisher’s Exact Test. Results revealed that most of the older patients were men (96.7%), aged 60–88 years (Mean = 71.57, SD = 7.75), with a smoking history (93.3%). The improvements were found in dyspnea (*p* < .01), its severity during activities (*p* < .01), and the quality of life (*p* = .04) among patients who attended the program. However, both groups did not have a different pulmonary function (*p* = .25) and the proportion of readmission within 28 days (*p* = .50). This study shows that the symptom management program can reduce dyspnea and severity during activities and improve the quality of life. Older people suffer from chronic obstructive pulmonary disease, especially when experiencing dyspnea. Therefore, it is crucial to have a symptom management program for older patients, especially a program developed to respond to changes in the aging process and the limitations of older people. This developed program was age-friendly to deal with symptoms and improve quality of life. However, this program should be explored in typical situations without the effects of the coronavirus disease (COVID-19) pandemic. In addition, more extensive population-based studies and randomized controlled trials should be adopted to increase credibility and ensure generalization.

*Clinical Trial Registration Number*: https://osf.io/6sj7y (October 4, 2021).

## Introduction

Chronic obstructive pulmonary disease (COPD) is a disorder in the lungs’ small airways. It leads to restriction and resistance to airflow in and out of the lungs, especially among older adults with their aging process related to organ and functional degeneration and long-term exposure to COPD risk factors^1^. Many processes, such as destroying parts of the lungs, secretions blocking the airway, and inflammation and swelling of the respiratory mucosa, narrow the airways, leading to breathing difficulties^1^. Based on world statistics, COPD is a significant public health problem and a primary disease affecting the quality of life, which causes suffering, disability, and mortality^2^. The statistics from all countries across the globe found that COPD is the third leading cause of death in the world in 2022^1,2^. According to statistics in Thailand in 2020, COPD was the sixth leading cause of death, with more than three million cases, an average of six per minute. In addition, more than one million patients are admitted to hospitals due to exacerbation, commonly found among older people, people with smoking habits, or those living in highly air-polluted areas^3^. In older people, it was also found that COPD was a significant disease causing disability and death. The mortality rate also substantially increased in this population, making this disease an area of concern for researchers and healthcare providers^1,2^.

## Background

Older adults are at risk for COPD of both the significant risk factors and changes in older adults according to the aging process^1,4^. The significant risk factors for COPD are smoking, pollution, chemical substances, and dust in the air^2,5^. Studies found that older people are exposed to the main risk factors for a long time due to inappropriate health behaviors, such as smoking and secondhand smoke. Moreover, their occupations cause them to be exposed to pollution, chemical substances, and dust in the air, causing many older people to suffer from COPD^5,6^. Other factors were the respiratory system and lung tissue changes with age^1,4^. In this regard, the decline in the respiratory structure among older people affects the strength of the respiratory muscles, ventilation balance, resistance to airflow, the retraction ability of the chest and lungs, and self-defense mechanisms of the respiratory tract, resulting in a decrease in lung volume, secretion retention, and an increase in the work of breathing^1,7–9^. Owing to these factors, the incidence of COPD in older people was higher than in other ages, and the GOLD stage of COPD among older patients is usually more advanced. Since the mean age of COPD diagnosis was 40 years, and the life expectancy of Thai people was 79, these patients often suffer from multimorbidity and polypharmacy burden along with living with COPD diagnosis for a longer time when compared with other age groups^1,2,5,6^.

Older people suffer from symptoms induced by the disease pathology when diagnosed with COPD. Older people have severe symptoms and many physical, mental, emotional, and social impacts. Regarding the physical aspect, the common symptom in older patients is dyspnea. It was found that older patients with COPD would experience dyspnea when exercising or doing activities. As for the psychological aspect, older patients suffer from shortness of breath or a feeling of dying. They cannot live everyday life like ordinary people, so it is necessary to limit daily routines, exercise, work, and activities that lower the quality of life of older patients with COPD^2,5^. In some cases, older patients cannot control or manage their symptoms, causing respiratory failure that quickly leads to disability and death. As for the emotional and psychosocial effects, it was found that the loss of existing abilities caused older adults to have less self-esteem and high perceived pressure, anxiety, fear, anger, or sometimes getting furious for no reason. In addition, depression can be found in older patients with COPD^1,10^.

Currently, care and treatment for patients with COPD are mostly symptomatic treatment, elimination of the cause or precipitating factor, and palliative care^5,11^. COPD treatment aims to relieve symptoms, prevent exacerbation, maintain pulmonary function, and promote quality of life. Disease preventions include inhalers and oxygen therapy in case of breathing difficulties. This treatment will start with an oxygen cannula at 1–2 L/min. In addition, studies have shown that a low oxygen supply during exercise enables patients to have better exercise tolerance. Symptoms exacerbation prevention can be done by reducing risk factors, such as smoking, secondhand smoke exposure, infection, and behaviors inappropriate with the disease. Pulmonary function maintenance focuses on avoiding risk factors, especially quitting smoking or changing occupations and exposure to precipitating factors, such as dust, smoke, and pollution, to stop or slow the pathology and disease progression. Finally, quality of life promotion is trying to maintain the ability to perform daily activities and socialize as before^1,5,11^. Because care and treatment for patients with COPD focus on self-care, symptom management, and behavioral modification, nurses play an essential role in providing care, advice, and encouragement to this population. Then older people can take care of themselves at home, carry out daily activities as usual, and have a good quality of life^5,11,12^.

The reviews by O'Donnell et al.^13^ and Volpato et al.^14^ have showed that interventions reducing inspiratory neural drive and regional lung hyperinflation and promoting breathing pattern and pulmonary blood flow can improve dyspnea and its psychological effects. These interventions included oxygen therapy, opiates, bronchodilators, inspiratory muscle training, combined exercise training, psychotherapy, self-management, relaxation therapy, music therapy, and pulmonary rehabilitation. Based on the literature review on intervention studies, researchers also developed many interventions for managing and caring for patients with COPD. However, these studies were conducted in adult patients or mixed-age groups. We realized that the pathogenesis, risk factors, and skills in perceiving information, learning, and understanding among older people might be limited and different from those of adults^15,16^. The aging process causes some degeneration of organ function and impairment, affecting health education for older people. These impairments include vision, hearing, attention, concentration, and less ability to remember information. Then, the provision of health education or knowledge needs to be specific in line with the limitations and requirements of older people^17,18^, and the previous interventions might be ineffective with older patients, especially Thai older patients who have specific lifestyles and contexts such as occupations relate to risk factors, foods and drinks, activities in daily, types of exercise and culture. Therefore, gerontology experts recommend that teaching older people should take a short time, be divided into sessions, and consist of explanations and practices. Practices must be simple and uncomplicated in a suitable place, and repeated teaching and training sessions should be provided. The teaching style considering the context and culture of older people should be concerned with using the proper media, large visible text, simple language, and age-friendly colors to be seen easily and clearly. In addition, teaching materials should be available for older people to take home and review^15,17,18^. To the best of our knowledge and based on our review, the existing nursing care methods or programs have not yet consistently responded to older people’s requirements and limitations. Consequently, developing a program specific to older people’s requirements and limitations is necessary.

## The study

### Aims

To study the effects of a symptom management program on selected health outcomes, including dyspnea and its severity during activities, pulmonary function, quality of life, and readmission rate within 28 days among older patients with COPD.

### Hypothesis

After attending the program, dyspnea, its severity during activities, pulmonary function, the quality of life, and the proportion of readmission within 28 days will be improved in older patients when compared with before attending the program and a control group.

### Sample size

The sample size was calculated using the equation of Schlesselman^19^ and a study by Chantaro^20^. The alpha level was set at 0.05 a priori with a power of 0.90 and an effect size of 0.768. Based on the calculation, 12 hospitalized older adults were needed for this study. However, 20% of participant dropouts were added to obtain an adequate sample. A sample of 15 participants for each group would meet the statistical criteria, and the total participants for this study were 30 hospitalized older adults.

### Design/methodology

In this quasi-experimental research with a two-group pretest–posttest design and a double-blind study, the participants were older adults aged 60 years and over, diagnosed with COPD by the physician using signs and symptoms, history of exposure to risk factors and spirometer test, and received treatment at the outpatient department of a medium-sized hospital in the northeastern region of Thailand. The participants were selected according to the inclusion criteria to assign the older patients to the experimental and control groups, totaling 15 patients each. The drawing was made to determine the order of study. The drawing result was to start conducting the study in the control group. After completing the outcome assessment process in this group, the experimental group conducted the study. The principal researcher provided the symptom management program, and the research assistant collected the data. Then, the results of the study were analyzed. In this regard, those who collected and analyzed the data did not know which participants were the experimental or the control groups to prevent bias in conducting the study and analyzing the results. Finally, there was no dropout for this study, so 30 patients were included for data analysis. In conducting this study, we confirmed that all process was performed in accordance with relevant guidelines/regulations and the Declaration of Helsinki, and informed consent was obtained from all participants and/or their legal guardians.

### Participants

#### Inclusion criteria

There were seven criteria including (1) Older men and women aged 60 years and over with COPD, (2) They are in the remission phase, (3) No signs and symptoms limiting their ability to practice exercises during this study, including low blood pressure, chest pain, and cardiac arrhythmia, (4) Have no depression, screened by a two questions (PHQ-2 or 2Q) screening test at the time of this study, (5) No cognitive impairment, assessed by the Thai Mental State Examination (TMSE), (6) No hearing and vision impairment and be able to communicate in Thai, and 7) Be voluntary and willing to cooperate in the study.

#### Exclusion & eliminate criteria

Patients who participated in a similar symptom management program within six months, experienced symptoms exacerbation during the program, and attended the program less than 80 percent were excluded from this study.

### Data collection

The principal researcher selected the participants according to the inclusion criteria, comprising older patients with COPD who visited the outpatient department at a medium-sized hospital in the northeastern region of Thailand from late June to September 2020. After screening and passing the criteria, the research assistants asked participants to participate in this study. They voluntarily participated in this study by signing the informed consent. To avoid cross-contamination of the program, the principal researcher randomly arranged the sequence of the study. As a result, the study was conducted earlier in the control group in June and July. This group received routine care, including vital signs and health assessment, review of signs and symptoms, treatments, and recommendations related to their health problems. After that, the study was conducted in the experimental group in August and September. The experimental group received the same routine care as the control group, plus the symptom management program. The research assistants collected data for the pretest before starting the program, and the posttest would be made 28 days after the completion of the program. Data collection continued until the required number of 30 patients was obtained, consisting of 15 patients in each group. The adhering record sheet monitored the adherence rate. The principal researcher phoned the older participants for 4 weeks, and their family members helped verify their adherence. Finally, all participants adhere to the program over 95%.

### Intervention

**Figure 1:**
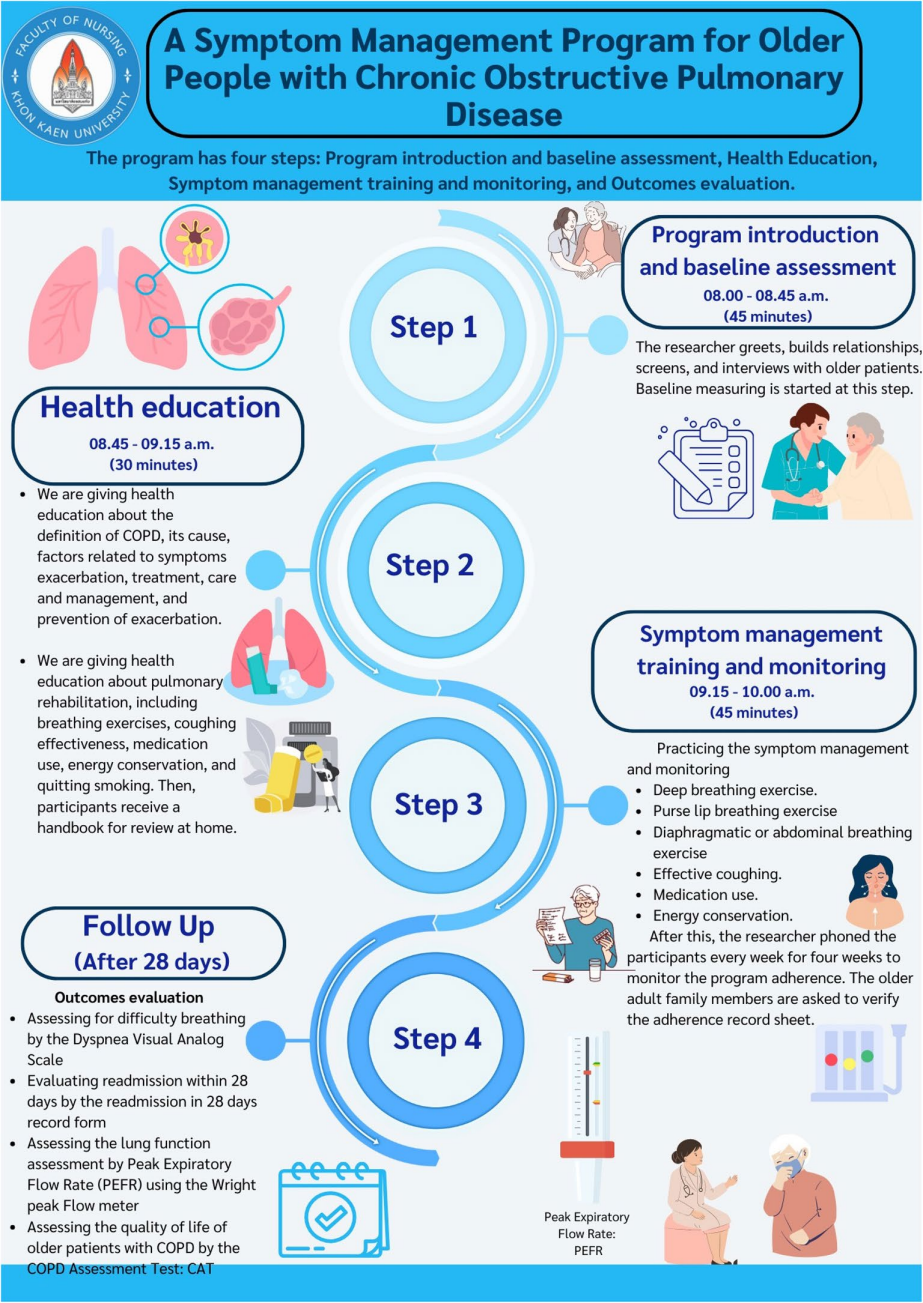
Demonstrate a step by step of a symptom management program for older people with chronic obstructive pulmonary disease.

The symptom management program for older patients with COPD is provided below**.** Moreover, a step-by-step of the program was also provided (Fig. 1).

1. We start the program with a program introduction and baseline assessment. The principal researcher and research assistant greeted the older adults, built relationships with them, and screened and interviewed them to be included in this study. Baseline measuring is started at this step before allowing the older patients to attend the program.
2. Teaching health education was based on the teaching plan to provide knowledge on COPD and self-management skills following the symptom management program provided by Dodd et al.^21^ This concept included symptom experience (What are the common and significant symptoms?), symptom management (What are the symptom management strategies?), and symptom outcomes (What is the patient's expected outcome?). Health education mainly consists of knowledge of COPD, factors related to exacerbation and prevention, treatment, dyspnea management, and pulmonary function promotion. In addition, pulmonary function promotion included breathing exercises, effective cough, medication use, energy conservation, and smoking cessation. The principal researcher provided health education individually for 30 min in the private room of the outpatient department. Then, patients had an opportunity to ask questions and receive more explanations, and a handbook was also given for this step. A handbook is a guidebook for providing health education, sizing 10 × 15 inches, totaling 15 pages, consisting of pictures and content. This handbook mainly used bold and large text, clear fonts and images, and age-friendly colors visible to older people, such as black, red, orange, and dark yellow, on a white background—the handbook's contents consist of definition, pathophysiology, complications, and treatment.
3. Symptom management training and monitoring is a practicing symptom management skill, i.e., self-management of dyspnea among older patients with COPD, consisting of breathing exercises, purse lip breathing, abdominal and diaphragmatic breathing, effective cough, medication use, and energy conservation. Three exercises, including the breathing exercise, energy conservation, and effective cough, will be practiced for 45 min. The practice will be done in a well-ventilated room with no noise. There are chairs to rest and staff to assist in practice. Then, the patients practiced at home for 4 weeks, and a checklist was provided and monitored by phone call.
4. The evaluation will be conducted 4 weeks after participating in the program. For this evaluation, the instrument included the Dyspnea Visual Analogue Scale (DVAS) based on the assessment form of Boonsawat^22^, the Peak Expiratory Flow Rate (PEFR), the COPD Assessment Test (CAT), and the readmission record form.

### Research instruments

1. Screening forms for assessing readiness included a depression screening form (2Q) with a sensitivity of 97.30% and a specificity of 45.60%^23^ and a Thai Mental State Examination (TMSE) with a sensitivity of 82% and a specificity of 70%^24^. The 2Q has two questions, and the response items were “Yes” or “No.” The TMSE includes 11 questions about orientation for time and place, registration, attention/calculation, recall, naming, repetition, verbal and written command, writing, and visuoconstraction. The participants will get the score when they respond with the right answer, and score rank from 11 to 30. Patients who answered “no” for both questions of 2Q and had a score of TMSE higher than 23 indicated they had no depression and cognitive impairment and were ready for this study.
2. The instrument for conducting the study was a symptom management program and handbook developed by the principal researcher from the literature review. First, the three experts, a medical professor specializing in COPD, a nurse professor, and a registered nurse specializing in caring for older patients with COPD, reviewed and provided recommendations for program development. After that, the principal researcher revised the program and handbook according to the experts' recommendations. Then, this program was implemented with five older patients with similar qualifications to the participants. We found the program suitable, understandable, and easy to follow, and it takes little time. In addition, the handbook is short, concise, and uses simple language, with large text and transparent images, which are easily readable and portable. Only two problems with the font size and three illustrations need to be clarified due to the file and printing quality. The principal researcher adjusted the font size to be larger and changed the image using the original file so that it did not get blurry when enlarged before using it in the actual study. The content validity index (CVI) from the three experts for this program was 1.0. This pilot was published in Thai and was entitled "Effects of symptoms management program on health outcomes among older people with chronic obstructive pulmonary disease, Nongwauso Hospital, Udonthani Province: A pilot study.
3. The research instrument for data collection included five instruments.
4. The Dyspnea Visual Analogue Scale (DVAS) is a highly accurate instrument COPD patients widely use. The concurrent validity was determined from the current condition (Criterion-Related Validity) to find the relationship between the measured scores and the instrument’s external criterion, namely, PEFR (Peak Expiration Flow Rate), which measures the maximal expiratory flow. Based on a study among 16 patients with asthma, the metric scores correlate with airway obstruction as 0.85^25^. The DVAS was a straight line 100 mm in length. The participants were asked to make a line indicating their dyspnea. The score rank from 0 to 100. The more the score, the more they suffer from dyspnea.
5. The assessment form for severity during activities was measured by requiring older patients with COPD to estimate the 5-level severity of the symptoms. This instrument was widely used in research and practice among patients with COPD. It was developed from the Modified Medical Research Council Dyspnea Score (mMRC) of the Global Initiative for Chronic Obstructive Lung Disease (GOLD)^26^. The reliability of the assessment form for severity during activities was 0.84. This assessment had one item with five severity levels (0–4) when 0 refers to no exertion during activity, but 4 indicates exertion when performing only activity daily.
6. The record of readmissions within 28 days is to record the number of times the older patients were admitted to receive treatment for exacerbation of dyspnea or COPD complications within 28 days after the study. The reason for readmission does not include treatment for other causes, such as falls and fainting due to low sugar levels. The three experts assessed this form, and CVI was 1.0. The number of readmissions was used to compare between groups. The more older patients are often readmitted, the poorer they can control their health condition.
7. The instrument for pulmonary function assessment is called the Wright Peak Flow Meter or Peak Flow Meter of GlaxoSmithKline Company. This instrument has been certified by industrial product standards and passed reliability testing before being used in the study. It was the accuracy of ± 5% of the readable value or ± 0.20 L/s^27^. As for the mouthpiece, it is a disposable type. Therefore, only one mouthpiece was used per patient. The result from a pulmonary function measured was a liter per second. The more liters per second they get, the more they have effective pulmonary function.
8. COPD Assessment Test (CAT) of GOLD 2019 was used to measure the quality of life among COPD Patients. This questionnaire was translated into Thai by GlaxoSmithKline Company and was widely used. According to the study by Chantha^28^, this assessment test has a reliability value of 0.87. The CAT included eight questions with six response items (0–5), of which 0 had no symptoms but 5 had severe symptoms. The score ranks from 0 to 40. The less they get the score, indicating a low impact of the disease on these patients, and they have a high quality of life.
9. The adherence record sheet was used to monitor the percentage of adherence in program activities. The record sheet includes all activities and a checklist. If older patients perform each activity in the program that they were trained at the hospital, the principal researcher checks on the provided list. This monitoring was done by a weekly telephone check and verified by the participant’s family members. The three experts assessed this form, and CVI was 1.

### Validity and reliability/rigor

The principal researcher developed the research program from a literature review. Three experts in three professional fields verified the content: a medical professor, a nurse professor, and a registered nurse. All experts were involved in the care and treatment of older adults and COPD. The feasibility and acceptability of the program were conducted among five older adults with the same health problem as the target group of this study. The results were already published in Thai. The outcome evaluator and statistician did not know whether they were in control or experimental groups. During the data collection, the research team carefully double-checked the data to ensure its correctness and completeness. Finally, the principal researcher registered a study protocol and strictly followed the protocol to reduce bias and increase the validity and reliability of this study.

#### Ethical considerations

The research received approval from the Khon Kaen University Ethics Committees for Human Research based on the Declaration of Helsinki and the International Conference on Harmonization (ICH) Good Clinical Practice Guidelines. The research project code was HE632103. Every research process adhered to the ethical principles of researching human subjects, including respect for persons, beneficence, and justice. The principal researcher explained the research project to the participants and gave time for decision-making and the right to make decisions without affecting the treatment process. Moreover, the patients could withdraw from the study whenever they wanted and continue to receive standard care for COPD patients. Finally, the control group would receive the symptom management program after finishing this study if needed.

#### Data analysis

Before generating the data analysis, assumptions were tested, including normality and homogeneity, and parametric and non-parametric statistics were applied based on the results. The participants' general characteristics data were analyzed using descriptive statistics. The mean scores between groups were compared using Independent T-test and Mann–Whitney U Test. The mean scores within groups were compared using the Dependent T-test and Wilcoxon Signed Ranks Test. The proportion of readmissions within 28 days was compared using Fisher’s Exact Test. Finally, an adjusted analysis was provided when finding the experimental and control group participants’ general characteristics and based line outcomes different. Fortunately, these differences were not found in this study, and an adjusted analysis was not conducted.

## Results

### General information

The participants consisted of 30 patients who ultimately attended the program, over 95%. Most of them were men (96.7%) aged between 60 and 69 years (46.7%) (Mean = 71.57, Max = 88, Min = 60, SD = 7.75). There were 100.0% Buddhists, 66.7% were married, and 86.7% graduated from primary school. The occupation of 60.0% was a farmer. Most families' monthly income was between 3000 and 10,000 baht, and 60.0% had adequate income/ no savings. Most of them used the universal coverage scheme (86.6%), and 96.7% had no problems with medical expenses. They were 93.3% smoking history, and 90.0% quit smoking (Table 1).

**Table 1:** Number, percentage, mean, minimum, maximum, and standard deviation of the samples classified by personal data.

Personal data	Control group (n = 15)	%	Experimental group (n = 15)	%	Total 2 groups (n = 30)	%
	Number		Number		Number	
Gender						
Men	14	93.3	15	100.0	29	96.7
Women	1	6.7	0	0.0	1	3.3
Age (years)	Mean = 71.67; Max = 87; Min = 60; SD = 7.81		Mean = 71.47; Max = 88; Min = 61; SD = 7.91		Mean = 71.57; Max = 88; Min = 60; SD = 7.75	
60–69	7	46.6	7	46.6	14	46.7
70–79	4	26.7	6	40.0	10	33.3
80 and over	4	26.7	2	13.4	6	20.0
Ethnicity: Thai	15	100.0	15	100.0	30	100.0
Religion: Buddhist	15	100.0	15	100.0	30	100.0
Marital status						
Married	12	80.0	8	53.3	20	66.7
Widowed	3	20.0	6	40.0	9	30.0
Divorced	0	0.0	1	6.7	1	3.3
Education level						
Primary school	13	86.7	13	86.7	26	86.7
Secondary school	2	13.3	2	13.3	4	13.3
Occupation						
Farmer	13	86.6	5	33.3	18	60.0
Employee	1	6.7	3	20.0	4	13.3
Unemployed	1	6.7	7	46.7	8	26.7
Average family income/month						
> 3000 Baht	4	26.7	1	6.7	5	16.7
3000–5000 Baht	4	26.7	5	33.3	9	30.0
5001–10,000 Baht	4	26.7	5	33.3	9	30.0
10,001 and Over	3	19.9	4	26.7	7	23.3
Income adequacy						
Adequate/have savings	3	19.9	5	33.3	8	26.7
Adequate/no savings	8	53.4	10	66.7	18	60.0
Inadequate/need to borrow	4	26.7	0	0.0	4	13.3
Health insurance						
Civil servant scheme	1	6.7	1	6.7	2	6.7
Social security scheme	1	6.7	1	6.7	2	6.7
Universal coverage scheme	13	86.7	13	86.7	26	86.6
Problems with medical expenses						
No problem	14	93.3	15	100.0	29	96.7
Few problems	1	6.7	0	0.0	1	3.3
Have you ever smoked?						
Yes	14	93.3	14	93.3	28	93.3
No	1	6.7	1	6.7	2	6.7
Are you currently smoking?						
Yes	1	6.7	2	13.3	3	10.0
No	14	93.3	13	86.7	27	90.0
Time of diagnosis (years)						
1–5 years	4	26.7	5	33.3	9	30.0
6 years and over	11	73.3	10	66.7	21	70.0
GOLD stage of samples						
GOLD 1	10	66.7	9	60.0	19	63.3
GOLD 2	5	33.3	6	40.0	11	36.7
Have you been hospitalized in the past 1 year?						
No	12	80.0	9	60.0	21	70.0
Yes	3	20.0	6	40.0	9	30.0
Current treatment						
Inhaler	11	73.3	9	60.0	20	66.7
Inhaler and oral medication	4	26.7	5	33.3	9	30.0
Inhaler, oral medication, and home oxygen therapy	0	0	1	6.7	1	3.3
Symptoms in the past 1 month						
Chronic cough	7	46.7	8	53.3	16	50.0
Thick phlegm	7	46.7	9	60.0	16	53.3
Shortness of breath	5	33.3	1	6.7	6	20.0
Dyspnea	7	46.7	10	66.7	17	56.7
The most common method used to treat dyspnea						
Oral medication	1	6.7	0	0	1	3.4
Inhaler	14	93.3	15	100.0	29	96.6

*n* number, *Min* minimum, *Max* maximum, *SD* standard deviation.

Most participants had been diagnosed with COPD for 6 years or longer (70.0%), and the GOLD stage was stage 1 (63.3%). In the past year, 70.0% had never been hospitalized. At present, most of them have received inhalers (66.7%). Most of the symptoms in the past month were dyspnea, thick phlegm, and chronic cough, which accounted for 56.7%, 53.3%, and 50.0%, respectively. The most frequently chosen method of managing dyspnea was the inhaler (96.6%) (Table 1).

### Effects of symptom management program on selected health outcomes among older patients with COPD

Before attending the program, there were no statistical differences in dyspnea, the severity of symptoms during activities, pulmonary function, and quality of life between the experimental and control groups (Table 2).

**Table 2:** Comparison of health outcomes among older people with chronic obstructive pulmonary disease in hospitals before and after the experiment between the control group and the experimental group.

Variable	Experimental group (n = 15)		Control Group (n = 15)		*t/Z*	*df*	*p*	MD	95% CI	
	Mean	SD	Mean	SD					Lower	Upper
*Before the program*										
Dyspnea	50.67	20.16	40.00	23.60	− 1.66^*M*^	–	0.09	10.66	−5.75	27.08
Severity During Activities	1.60	0.91	1.73	1.28	− 0.04^*M*^	–	0.96	− 0.13	− 0.96	.69
Pulmonary Function	54.74	15.73	66.55	19.18	1.84	28	.007	− 11.80	− 22.93	1.31
Quality of Life	17.66	7.87	12.73	12.73	− 1.72^*M*^	28	0.96	4.93	− 0.93	10.79
*After the program*										
Dyspnea	32.00	16.12	58.67	24.16	− 2.98^*M*^	–	< .01	26.66	11.30	42.03
Severity During Activities	0.80	0.56	1.90	1.22	− 2.86^*M*^	–	< .01	− 1.13	− 1.84	− .42
Pulmonary Function	58.47	14.19	65.19	17.30	1.16	28	0.25	− 6.71	− 18.55	5.11
Quality of Life	8.40	5.67	13.60	7.62	2.11^*M*^	28	0.04	− 5.20	− 10.22	− 0.17

*n* number, *SD* standard deviation, ^M^ Mann–Whitney U Test, *MD* median, *CI* confidence interval.

After attending the program, we found the difference in dyspnea (Z = − 2.98, *p* < 0.01), the severity of symptoms during activities (Z = − 2.86, *p* < 0.01), and the quality of life (t28 = 2.11, *p* = 0.04) between experimental and control groups. However, pulmonary function was not different (t28 = 1.16, *p* = 0.25) (Table 2).

After attending the program, dyspnea (Z = − 3.34, *p* < 0.01), the severity of symptoms during activities (Z = 5.52, *p* < 0.01), the pulmonary function (t14 = − 5.13, *p* < 0.01), and quality of life (t14 = 10.15, *p* < 0.01) were improved in the experimental group (Table 3). However, these improvements were not found in the control group (Table 3).

**Table 3:** Comparison of health outcomes among older people with chronic obstructive pulmonary disease in hospitals before and after the experiment within the control group and the experimental group.

Variable	Before the Experiment (n = 15)		After the Experiment (n = 15)		*t/Z*	*df*	*p*	95% CI	
	Mean	SD	Mean	SD				Lower	Upper
*The experimental group*									
Dyspnea	50.67	20.16	32.00	16.12	− 3.34^*w*^	–	< 0.01	− 22.65	− 12.01
Severity During Activities	1.60	0.91	0.80	0.56	− 3.20^*w*^	–	< 0.01	1.11	0.49
Pulmonary Function	54.74	15.73	58.47	14.19	− 5.13	14	< 0.01	− 5.28	− 2.17
Quality of Life	17.66	7.87	8.40	5.67	10.15^*w*^	14	< 0.01	7.30	11.22
*The control group*									
Dyspnea	40.00	23.60	58.67	24.16	0.61	14	.54	− 5.95	3.28
Severity During Activities	1.73	1.28	1.90	1.22	− 1.34^*w*^	−	.18	− 0.51	0.11
Pulmonary Function	66.55	19.18	65.19	17.30	1.43	14	0.17	− 0.67	3.40
Quality of Life	12.73	12.73	13.60	7.62	− 1.99^*w*^	14	0.06	− 1.79	0.06

*n* number; *SD* standard deviation; ^w^ Wilcoxon Signed Ranks Test; *CI* confidence interval.

Finally, we found one older patient (6.7%) in the control group and no older patients in the experimental group visiting the hospital within 28 days. After using Fisher’s Exact Test, it was found that the proportion of readmissions within 28 days among older patients in control and experimental groups was not different (*p* = 0.50) (Table 4).

**Table 4:** Comparison of number and percentage of the samples of older people with chronic obstructive pulmonary disease who were re-admitted within 28 days after receiving the symptom management program for older people with chronic obstructive pulmonary disease.

Re-admission within **28** days after discharge	The control group (n = 15)		The experimental group (n = 15)		Total 2 groups (n = 30)		*p*
	Number (Persons)	%	Number (Persons)	%	Number (Persons)	%	
Yes	1	6.7	0	0.0	1	0.3	0.50
No	14	93.3	15	100.0	29	99.7	

*n* *Number.*

## Discussion

Findings confirmed that the symptom management program could relieve dyspnea and severity during activities. The capacity imbalance theory can be used to explain the physiological bases for reducing dyspnea and its severity^13,14^. Dyspnea is caused by a breakdown in the regular interaction between the respiratory system's dynamic response and the inspiratory neuronal desire to breathe. By lessening the neuromechanical dissociation of the respiratory system, therapeutic therapies such as bronchodilators, exercise training, ambulatory oxygen, inspiratory muscle training, and opiates can alleviate dyspnea^13^, meaning successful management needs a combination of interventions^14^. This program included several breathing exercises promoting lung function and respiratory muscle. Breathing exercises can also promote ventilation and gas exchange. Health education and self-management prevent the patient from engaging in unhealthy behaviors that might stimulate symptom exacerbations; meanwhile, energy conservation helps them relieve fatigue. Finally, the effective cough and medication use included in this program supports the patient in clearing the airway from secretion and promoting bronchodilatation, resulting in dyspnea and its severity reduction.

Moreover, this program helped relieve dyspnea and severity because the principal researcher developed the program based on the literature review and evidence-based analysis. This symptom management program integrated multiple activities, including teaching, practicing, and giving a handbook for reviewing, which all jointly stimulated learning skills. In addition, activities in the program were carried out step by step based on the symptom management model of Dodd et al.^21^, consisting of symptom experience, symptom management strategies, and symptom outcomes. The symptom experience—older patients mainly experienced dyspnea, perceiving the symptom as severe and life-threatening. Symptom management strategies –The most important was to give inhalers and breathing therapies. This program showed a tutorial on giving inhalers, and the handbook clearly explained the processes with illustrations. The management outcomes showed that dyspnea and its severity during activities substantially decreased. Maricoto et al.^29^ found that a program that taught older patients how to use inhaler devices would enable patients to use them accurately and effectively and help significantly reduce COPD exacerbations, especially dyspnea.

The program’s design in this study is specific to the requirements and limitations of older people, so it helps older people learn quickly and achieve positive outcomes. The principles of health education or teaching for older people need to apply multiple strategies and adjust strategies to meet the requirements of older people to enhance teaching efficiency^15,16^. Older people have different characteristics from adults^4^, so they require specific care. The accessibility to older people must be specific and consistent with their limitations, such as changes in vision and hearing, reduced energy reserves, easy fatigue, and a decrease in cognitive ability, i.e., having less concentration and reduced memorization ability^17,18^. As a result, previous programs may have limitations and may not be suitable for older people^30^.

Therefore, this symptom management program was designed considering font size, illustrations, and color. Moreover, this provided knowledge and practice related to the Thai context and culture. Teaching procedures and periods were not too long. The stimuli transmitted through the five senses, namely, the ears, eyes, nose, tongue, and skin, produce more learning outcomes than stimuli transmitted through one sense only. The practice helped turn the abstract into concrete for better understanding^15,18^. As older adults had reduced memorization ability, the program was designed to have practices to enhance skills and ability to manage the symptoms independently. Moreover, repetitive self-practice would enable practitioners to learn effectively, understand reasons, and achieve sustainable learning^18^. Participation in the teaching and practice process would promote learning, memorization, and practicing with confidence^16,18^. As a result, the older patients who participated in the program had less dyspnea and severity during activities. Accordingly, Chantaro's study of the effects of a self-management program on dyspnea among patients with COPD revealed that their symptoms after attending the self-management program were better than those who received routine nursing care^20^.

This study found that although pulmonary function in the experimental group after participating in the program was significantly better than before joining the program, it was not different from that of the control group. This program included pulmonary function promotion and dyspnea self-management; however, the program's short duration might not clearly demonstrate the significant results between the two groups. This result is because pulmonary function improvement or rehabilitation requires a certain period to see the improvement. As shown in this study, the duration was approximately 4 weeks, so it caused minor differences. The results of this study differ from the study of Chouythoa, which explored the effects of a self-management program for 6 weeks, which was 2 weeks longer than this study^31^.

The symptom management program for older patients with COPD encouraged the patients who participated in the program to have a better quality of life. Older patients who received the symptom management program had quality of life scores of less than 10, which indicated a low impact of the disease on these patients. Proper management of exacerbations coupled with the promotion of pulmonary function is essential to improve the patient's quality of life as it would help reduce the symptoms of dyspnea and increase the ability of older patients to do daily activities as usual. This newly developed program was designed to enable older people to manage dyspnea, improve pulmonary function, and prevent exacerbations. If the pulmonary function could be enhanced, the severity of dyspnea decreased, and the incidence of exacerbations could be controlled, older patients could live everyday life and have a better quality of life. Accordingly, Duangseang's study on managing dyspnea among older patients with COPD found that patients who could effectively manage dyspnea would achieve high happiness scores and life satisfaction^10^. The study by Xiao and Zhuang found that regular physical activity/exercise by walking helped raise functional capacities, such as walking endurance, fatigue, pulmonary function, shortness of breath, mental health, and quality of life^32^. Maricoto et al. conducted a systematic review and meta-analysis of eight studies on older people. They concluded that the most powerful practice to reduce dyspnea exacerbations was the practice of using an inhaler and simulation equipment. After attending the program, the rate of exacerbation was reduced. At the same time, the quality of life tended to increase after attending the program^33^.

This study found no difference in readmission rates. Older patients who attended the program had no readmissions within 28 days, but only one older patient who received routine care was readmitted. The results of this study are similar to Li et al.’s^34^ study, which found that only one patient who received the triple therapy program had an exacerbation. Hung’s study on the effects of a hospital-to-home transition care program revealed that a program could help reduce the readmission rate^35^. The results mentioned above differed from this study, possibly because the hospital-to-home transition care program took longer, had more participants (212 persons), and used multidisciplinary teams to care for patients, resulting in apparent differences between the experimental and control groups. However, our study found no differences, possibly due to the small sample size and short study period. In addition, during the COVID-19 outbreak, this situation may cause older patients to be worried about receiving services as they were afraid to be at risk of contracting COVID-19 from hospitalization. Then, they did not decide to visit the hospital if they did not experience the worst symptoms. Another explanation was a policy to prevent the spread of COVID-19 by giving medication or receiving treatment at a sub-district health-promoting hospital instead of visiting a hospital^36,37^. Therefore, this may affect decision-making in receiving treatment at the hospital.

## Limitations

This study was conducted on mild and moderate-severity patients (GOLD levels 1 and 2) during the remission phase, not other groups with high and very high severity (GOLD levels 3 and 4), who often suffer and burden along with a longer time living with COPD diagnosis. Therefore, the study could not represent the entire population and limited use of the research findings in higher severity groups. Participants who attend this program have no hearing and visual impairment. These inclusion criteria make the participants less representative of older patients and limit generalization. In addition, almost all of the participants in this study were men. Therefore, it may not reflect the study’s results in the female population. In this case, future studies should be conducted among older women likely to be exposed to secondhand smoke. Finally, the small sample size, the short study period, and the COVID-19 epidemic may cause older patients to worry about deciding to receive services at the hospital. Therefore, this study could not show an apparent difference in readmission rates.

## Conclusion

The study on the effects of this symptom management program among older patients with COPD was conducted on 30 older patients. There were 15 patients in the experimental group receiving the symptom management program and 15 patients in the control group receiving routine nursing care. Outcomes were measured after attending the program for 28 days. The results revealed that the symptom management program could reduce dyspnea and its severity during activities and increase the quality of life. Therefore, the program should be applied to maximize efficiency and effectiveness in caring for older patients with COPD. However, this study may only partially reflect results due to the COVID-19 outbreak, which affects the healthcare service policies and the decision-making of older patients to receive healthcare services in case of an exacerbation. Therefore, this program should be explored in typical situations again, and outcomes should be measured over a more extended period. In addition, more extensive population-based studies and randomized controlled trials should be adopted to increase credibility and ensure generalization.

## Data Availability

The datasets generated and/or analyzed during the current study are not publicly available due to prohibited laws (and/or rules, regulations, and contracts). However, they are available from the corresponding author upon reasonable request.
